# [Retracted] Long non-coding RNA KCNQ1OT1 promotes nasopharyngeal carcinoma cell cisplatin resistance via the miR-454/USP47 axis

**DOI:** 10.3892/ijmm.2024.5355

**Published:** 2024-02-06

**Authors:** Feng Yuan, Zhiping Lou, Zhifeng Zhou, Xiaojun Yan

Int J Mol Med 47: 54, 2021; DOI: 10.3892/ijmm.2021.4887

Following the publication of this paper, it was drawn to the Editor's attention by a concerned reader that the colony formation assay data shown in Figs. 4C and 6D and the Transwell migration and invasion assay data shown in Figs. 4D, 6E and 6F were strikingly similar to data appearing in different form in other research articles written by different authors at different research institutes that had either already been published, or were submitted for publication at around the same time.

Owing to the fact that contentious data in the above article had already been published elsewhere prior to its submission to *International Journal of Molecular Medicine,* the Editor has decided that this paper should be retracted from the Journal. The authors were asked for an explanation to account for these concerns, but the Editorial Office did not receive a reply. The Editor apologizes to the readership for any inconvenience caused.

